# Prophylactic effect of the traditional Chinese medicine formula *danxiong* granules (TDX105) on hand–foot skin reaction associated with the antitumor targeted drug regorafenib: a randomized, double-blind, placebo-controlled trial

**DOI:** 10.3389/fphar.2025.1641477

**Published:** 2025-11-06

**Authors:** Shuang Yu, Xinrui Hu, Yaqin Tan, Caixia Wang, Zhenyu Shao, Ying Xiao, Hailong Liu, Jing Lv, Sheng Li, Xuan Jiang, Lingzhi Zeng, Aiping Tian

**Affiliations:** 1 National Cancer Center/National Clinical Research Center for Cancer/Cancer Hospital, Chinese Academy of Medical Sciences and Peking Union Medical College, Beijing, China; 2 Baotou Cancer Hospital, Baotou, Inner Mongolia, China; 3 Shandong Provincial Hospital Affiliated to Shandong First Medical University, Jinan, Shandong, China; 4 Qilu Hospital of Shandong University, Jinan, Shandong, China; 5 The First Affiliated Hospital of Chongqing Medical University, Chongqing, China; 6 Chenzhou No. 1 People’s Hospital, Chenzhou, Hunan, China; 7 The Affiliated Hospital of Qingdao University, Qingdao, Shandong, China; 8 Department of Medical Oncology, The Affiliated Cancer Hospital of Nanjing Medical University and Jiangsu Cancer Hospital and Jiangsu Institute of Cancer Research, Nanjing, Jiangsu, China; 9 The Second Affiliated Hospital of Chongqing Medical University, Chongqing, China; 10 Jiu Jiang No. 1 People’s Hospital, Jiujiang, Jiangxi, China

**Keywords:** hand-foot skin reaction, randomized controlled clinical trial, regorafenib, TDX105, traditional Chinese medicine formula

## Abstract

**Background:**

Hand–foot skin reaction (HFSR) is the most common side effect of the antineoplastic drug Regorafenib. It severely affects patients’ quality of life, and no clear treatment is currently available for the condition. In preliminary clinical studies, the traditional Chinese medicine compound Danxiong Granules (TDX105) has shown significant therapeutic benefit for HFSR. This study aimed to evaluate the prophylactic effect of TDX105 for HFSR.

**Methods:**

A total of 137 colorectal cancer patients scheduled for Regorafenib treatment were randomly assigned in a 2:1 ratio to a treatment group (n = 91) and a control group (n = 46), which received basic treatment (topical urea ointment) plus TDX105 or placebo, respectively, for 8 weeks. Follow-up was conducted until tumor regression or Regorafenib discontinuation. The primary study endpoint was the incidence of HFSR within 8 weeks.

**Results:**

The total incidence of HFSR was markedly lower in the treatment group than in the control group (76.1% vs. 53.8%), particularly for grade 3 HFSR (7.7% vs. 19.6%, *p* = 0.041; absolute risk difference: 11.87%, 95% confidence interval: −0.01–0.25). Moreover, TDX105 significantly delayed the median onset time of HFSR (25 vs. 11 days, *p* < 0.001) and decreased the durations of grades 2 and 3 HFSR (grade 2: 12 vs. 22 days; grade 3: 5 vs. 13 days, *p* < 0.01). The rate of Regorafenib dose reduction due to HFSR was significantly lower in the treatment group (1.10% vs. 19.57%, *p* < 0.05). Importantly, the HFSR continuation rate was 0% in the treatment group, compared to 10.87% in the control group. Although tumor control rates were similar in both groups, progression-free survival was significantly improved in the treatment group (3.2 vs. 2.5 months, *p* = 0.048).

**Conclusion:**

TDX105 significantly reduced the incidence and severity of Regorafenib-induced HFSR. This finding lends support to the use of TDX105 for prevention of HFSR.

**Clinical trial registration:**

NCT05289726.

## Introduction

Due to their inhibitory effects on the growth and spread of cancer cells, targeted drugs are generally better tolerated than traditional chemotherapy drugs and are increasingly used in cancer therapy. However, they often lead to dermatological toxicities. Some multi-kinase inhibitors, including sorafenib, exert their effects by targeting angiogenesis and proliferation in tumor cells. Consequently, these drugs can cause vascular damage due to the inhibition of vascular endothelial growth factor receptor and platelet-derived growth factor receptor and then can induce skin lesions due to their leakage from blood vessels, resulting in hand–foot skin reaction (HFSR) (Chen et al., 2022). This is the most common side effect induced by multi-kinase inhibitors (Reyes-Habito and Roh, 2014), and Regorafenib is associated with an even higher incidence of HFSR than other multi-kinase inhibitors (Wang et al., 2020). In two phase III clinical trials, Regorafenib (Stivarga) was found to significantly extend the survival of patients with metastatic colorectal cancer. The global phase III CORRECT study showed that the median overall survival (OS) of patients in the Stivarga group was significantly extended to 6.4 months (Grothey et al., 2013), while the Asian regional CONCUR study indicated that, relative to that of the placebo group, the median OS of patients in the Stivarga group was extended to 8.8 months (Li et al., 2015). However, the incidence of drug-related adverse events caused by Regorafenib treatment also was higher (97% vs. 46%) than in the placebo, with fatigue, HFSR, diarrhea, and hypertension being the most common (≥30%), mostly of grade 1 or 2. Moreover, another study reported that adverse events generally occurred early in Regorafenib treatment, and the incidence tended to decrease over time (Yamaguchi et al., 2019).

HFSR, specifically, is one of the most prominent clinical toxic side effects of Regorafenib, with a reported incidence rate as high as 61%, of which 20% is grade 3 HFSR. In the Asian population, the incidence of HFSR was reported to reach up to 74% (Li et al., 2015). This condition manifests mainly as painful erythema, swelling, and tingling on the palms and soles, and it leads to skin desquamation, blistering, ulceration, and even infection in severe cases (McLellan et al., 2015). These presentations severely affect the implementation of the antitumor treatment plan, which indicates that patients who are very likely to benefit from Regorafenib treatment may face the risk of dose reduction and discontinuation due to skin toxicity reactions in the early stages of treatment.

Previous research has shown that the clinical severity of HFSR may be related to the efficacy of targeted drug therapy (Miller et al., 2014). In one study, the severity of HFSR was associated with the median OS of patients and patients with higher-grade HFSR had a better prognosis than those with lower-grade HFSR (Kobayashi et al., 2019). Accordingly, patients who exhibit early HFSR tended to experience relatively better OS benefits. Therefore, early preventive intervention to ensure patients’ good tolerance to Regorafenib may help to improve the overall therapeutic effect, thereby resulting in better tumor control (Yin et al., 2018).

Currently, the prevention and management of HFSR primarily rely on a comprehensive set of measures such as patient education to recognize early symptoms and skin protection to reduce skin friction and pressure (Grothey et al., 2014). The treatment of HFSR involves the use of topical corticosteroids to alleviate inflammation and vitamin E and traditional Chinese medicine (TCM) to reduce symptoms. Unfortunately, these treatments have shown limited effectiveness, and dose adjustments or interruptions of Regorafenib may still be necessary when required (Chanprapaph et al., 2016). One study found that after two cycles of treatment with topical clobetasol propionate for regorafenib-induced HFSR, only 13% of patients did not experience further occurrence or worsening of HFSR (Jatoi et al., 2021). Currently, no specific drug has been clearly designated for the treatment of skin toxicities related to targeted drugs. The current common practice is based on “best practices,” which lack evidence-based support. As a result, many patients still face the risk of interruption or discontinuation of antitumor treatment due to skin toxicities. Therefore, there is an urgent need to identify more effective and safer treatment options. In this context, the TCM compound Danxiong Granules (TDX105), with its unique herbal combination and long-term clinical application experience, has demonstrated potential therapeutic advantages.

TDX105 consists of five Chinese medicinal herbs: *Ligusticum chuanxiong* Hort [Apiaceae; *Conioselinum anthriscoides* “Chuanxiong”] (Wang, et al., 2025), *Carthamus tinctorius L.* [Asteraceae; safflower] (Bai, et al., 2025), *Geranium wilfordii* Maxim. [Geraniaceae; *Geranium wilfordii*] (He, et al., 2022), *Phellodendron amurense* Rupr. [Rutaceae; Phellodendri amurensis cortex] (Sun, et al., 2019), and *Paeonia* × *suffruticosa* Andrews [Paeoniaceae; Moutan cortex] (Liu, et al., 2023). The aqueous solution of TDX105 is used externally for treating skin toxicities related to targeted drugs, and evidence of its clinical efficacy has been observed over more than 10 years of its application. A previous prospective, randomized, double-blind clinical study focused on skin damage associated with antitumor targeted drugs and found that the efficacy of a 10-day treatment of HSFR with TDX105 was 95.45%, which was markedly higher than the corresponding value of 27.27% for current common methods used in the control group. Pain relief was observed within 1–2 days, and within 3–10 days, TDX105 substantially alleviated pain, rash, blisters, edema, ulcers, and other skin lesions (Tian et al., 2017).

Therefore, in the present study, we evaluated the prophylactic efficacy of TDX105 for preventing regorafenib-induced HFSR in colorectal cancer patients, using placebo as the comparator. The study also monitored the rates of dose reduction, interruption, or discontinuation of Regorafenib due to skin reactions, as well as the impact of the preventive intervention on the tumor control rate and progression-free survival (PFS). The overall aim was to assess the feasibility of using this TCM to reduce the side effects of targeted drug therapies, with a view to improving compliance with cancer treatments in support of the application of optimal targeted drug dose levels in comprehensive cancer treatment protocols.

## Methods

### Study design and study population

This was a randomized, double-blind, placebo-controlled clinical trial conducted from February 2021 to January 2023 and designed to evaluate the prominent skin toxicity associated with regorafenib (Stivarga). Patients undergoing Regorafenib treatment for the first time were enrolled. Based on previous clinical research results and experience, we expected the treatment protocol for the experimental group to yield positive outcomes, thereby offering the maximum potential benefits to participants. Moreover, a larger sample size in the experimental group enhanced the statistical power to detect treatment effects. Therefore, we employed block randomization to randomly assign patients to the experimental and control groups in a 2:1 ratio. Random allocation was performed using an online computer-generated random number generator. Patients’ numbers were first inputted to produce a sequence of random assignments, and then each patient was allocated to the experimental or control group based on an allocation number. An encoding system was implemented to conceal group information, which was known only to the personnel responsible for encoding. The results of random allocation were placed in opaque envelopes to ensure that the research team and patients remained unaware of group assignments throughout the trial period. Patients in both groups were given urea ointment as a basic preventive measure. The experimental group also received the TCM TDX105, while the control group was treated with placebo granules.

The primary study endpoint was the incidence of all grades of HFSR, while the secondary endpoints were the duration of HFSR at all grades; rate of dose reduction, extent of interruption, or discontinuation of Regorafenib; tumor control rate; and PFS. The trial was designed to determine whether the external use of the tested TCM reduces the incidence and severity of HFSR, improves patient compliance with Regorafenib treatment, and aids in the implementation of optimal dosing in tumor treatment regimens.

This project was registered in the ClinicalTrials.gov Protocol Registration and Results System (registration No: NCT05289726). Ethical approval for the study was obtained from the National Anticancer Drug GCP Central Ethics Committee (Approval No: 21/426-3097, issued on October 14, 2021). This study was conducted in accordance with all relevant ethical guidelines, and all patients submitted a signed informed consent form.

The study participants included inpatients and outpatients from the Cancer Hospital of the Chinese Academy of Medical Sciences, as well as cancer patients from eight other hospitals.

#### Inclusion criteria

Patients were included in the study if they met the following criteria: (1) clinical diagnosis of colorectal cancer; (2) planned treatment with Regorafenib (Stivarga) for the first time; (3) Performance Status (PS) score ≤3 points; (4) age ≥18 years; and (5) good understanding of the objectives of the study, agreement to accept the treatment, and provision of written informed consent.

#### Exclusion criteria

Patients were excluded from the study if they met any of the following criteria: (1) treatment with other targeted drugs such as Sorafenib or Sunitinib, chemotherapy drugs such as Capecitabine or Doxorubicin, or radiotherapy on the hands and feet, which may affect skin reactions; (2) presence of skin diseases such as eczema, psoriasis, or hand and foot dermatophytosis on the exposed skin areas (i.e., hands and feet); (3) presence of any allergic skin disease; (4) presence of intellectual or mental disability, or inability to communicate verbally or describe the symptoms experienced; (5) lack of cooperation in continuing treatment during the study; or (6) sudden deterioration of clinical condition that affected or interfered with the study.

### Therapeutic regimen

This study was planned to initiate concurrent use of the TCM TDX105 at the start of Regorafenib treatment. The experimental group was given TDX105, whereas the control group received a placebo granule that had comparable color and appearance to TDX105. The placebo was made by blending dextrin and food coloring agents. Both the TCM TDX105 and placebo were products of China Resources Sanjiu Medical and Pharmaceutical Co., Ltd. We obtained the Consensus statement on the Phytochemical Characterisation of Medicinal Plant extracts (ConPhyMP) checklist by using the ConPhyMP tool, which defines the best practices for reporting the starting plant materials (Heinrich et al., 2022). The dose used was 4.5 g (granule weight of Chinese herbal decoction pieces) at each application, dissolved in warm water and diluted to 500 mL. Starting from the first day of enrollment, patients soaked their hands and feet in the solution at a temperature of approximately 25 °C–30 °C for 30 min, once every evening, until the end of the first 8 weeks of Regorafenib (Stivarga) treatment. After the preventive intervention, patients were followed up until tumor progression and discontinuation of Regorafenib (Stivarga). The follow-up was performed by dedicated personnel, twice per week during the first month of treatment, weekly during the second month, and monthly after 2 months, until tumor progression occurred and the follow-up was terminated. The grading criteria for HFSR was based on the Common Terminology Criteria for Adverse Events (CTCAE) (National Cancer Institute, 2009) version 4.0 (Table 1). The grading and diagnosis of HFSR lesions were done by two researchers upon reaching a unanimous decision. The response to tumor treatment was evaluated every 2 months using the Response Evaluation Criteria in Solid Tumors (RECIST) criteria (Eisenhauer et al., 2009).

**TABLE 1:** NCI CTCAE grading of HFSR.

NCI grade	Description
Grade 1	Mild skin changes or dermatitis (such as erythema, edema, or hyperkeratosis), without pain
Grade 2	Skin changes such as peeling, blisters, bleeding, edema, or hyperkeratosis, with pain and limitations in functional activities of daily living: cooking, shopping, making phone calls, handling money, etc.
Grade 3	Skin changes such as peeling, blisters, bleeding, edema, or hyperkeratosis, with pain, and limitations in self-care and activities of daily living: bathing, dressing, eating, using the toilet, and taking of medication

### Statistical analysis

Statistical analysis for the primary endpoint, the incidence of HFSR was done using a test for superiority to compare two independent sample rates. The primary results are reported as absolute risk difference (ARD) with 95% confidence interval (CI). The durations of skin reactions at various grades were compared using t-test or rank-sum tests, while the rates of dose reduction, interruption, or discontinuation of Regorafenib were compared using chi-square (χ^2^) test. The tumor control rate and PFS were compared using the log-rank test, and multivariate regression analysis was employed to control for potential confounding factors that might affect the incidence of HFSR and other endpoints. All statistical analyses were performed using SPSS 27.0 software. Differences were considered statistically significant at *p* < 0.05.

## Results

### Sample size calculation and patients’ information

In the calculation formula for the test of superiority based on two-group rates, the overall incidence of HFSR in the experimental group was assumed to be 40%, while that in the control group was 70%. With an alpha level (α) of 0.025, a beta level (β) of 0.2, and a superiority margin of −5%, the calculated sample size required for the experimental group was 83, while that for the control group was 42. Accounting for a dropout rate of 10%, the total sample size needed for the study was calculated to be 138, with 92 in the control group and 46 in the experimental group.

**FIGURE 1:**
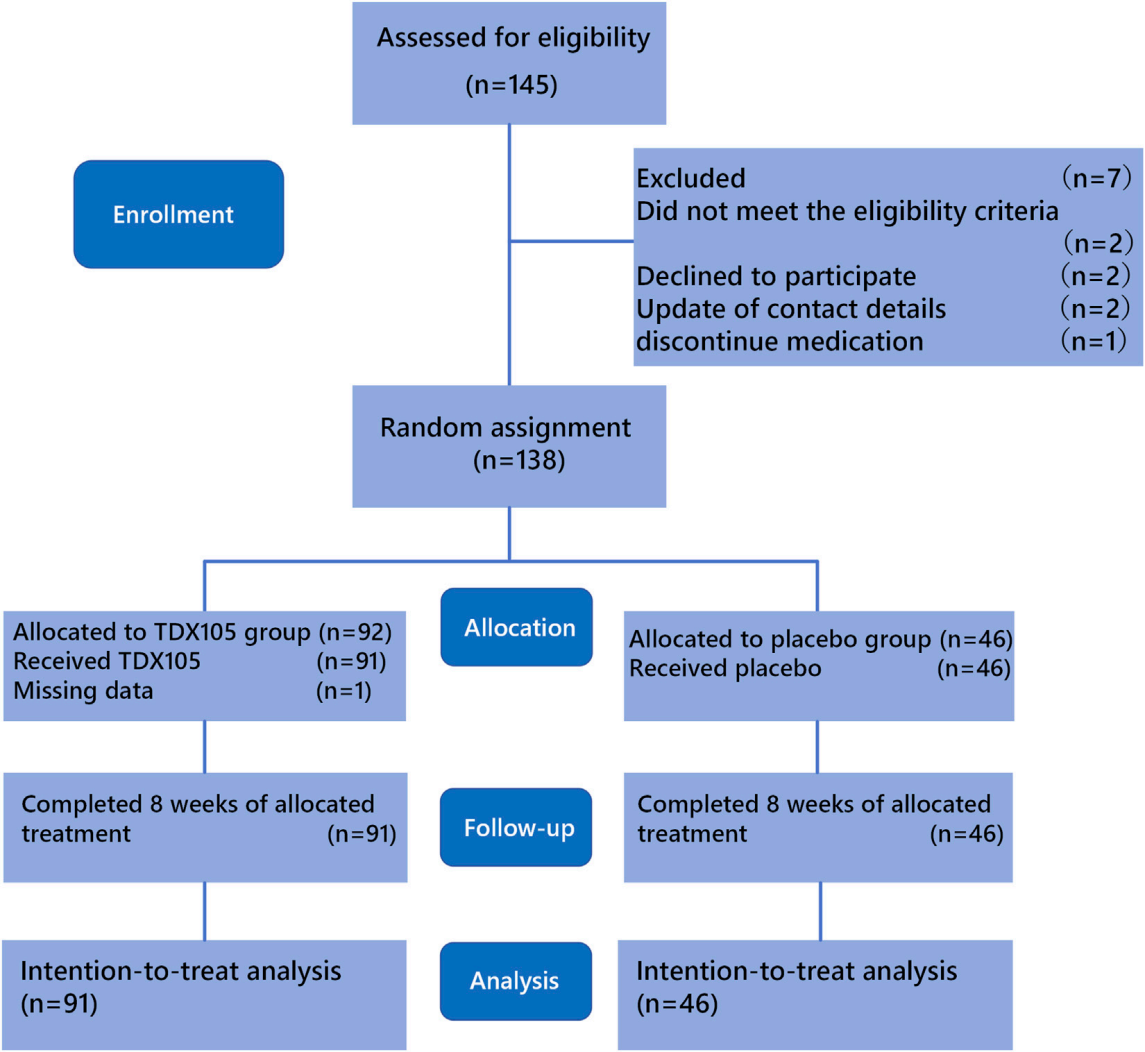
CONSORT diagram of patient inclusion and exclusion.

Between March 2022 and May 2024, according to the inclusion and exclusion criteria for this study, a total of 145 colorectal cancer patients from the Cancer Hospital of the Chinese Academy of Medical Sciences and eight other hospitals were enrolled in the clinical trial. During the follow-up process, 8 patients were withdrawn due to unwillingness to cooperate, cessation of medication, change of contact information, and other reasons, and were not included in the analysis. Therefore, 137 patients ultimately completed 8 weeks of treatment and were included in the analysis. All 137 were stage 4 cancer patients who had undergone chemotherapy and were randomly assigned to receive TDX105 (n = 91) or placebo (n = 46) (Figure 1).

In the experimental group and control group, 17 and 9 patients, respectively, received Regorafenib at a dose of 160 mg/day. However, due to the high incidence of dose reduction and treatment interruption caused by Regorafenib-induced side effects, such as liver damage, diarrhea, hypertension, mucositis, and skin lesions, the dose of Regorafenib was reduced for all other patients. Ultimately, the other 74 patients in the experimental group and 37 patients in the control group took Regorafenib at a lower dose of 120 mg/day. All patients completed 8 weeks of treatment, with 66.4% receiving TDX105% and 33.6% receiving placebo treatment. The baseline demographic and clinical characteristics of patients in the two groups were comparable, as shown in Table 2.

**TABLE 2:** Baseline characteristics of patients (n = 137).

Characteristic	All (n = 137)	TDX105 group (n = 91)	Placebo group (n = 46)
Age, years, median (IQR)	63 (57–70)	63 (58–70)	62 (53–70)
Sex, n (%)			
Female	37 (27.1)	24 (26.4)	13 (28.3)
Male	100 (72.9)	67 (73.6)	33 (71.7)
Regorafenib dose, n (%)			
160 mg	26 (18.9)	17 (18.6)	9 (19.5)
120 mg	111 (81.0)	74 (81.3)	37 (80.4)
Regorafenib mean actual dose, mg/day, mean (SD)	116 (18)	117 (18)	116 (19)

### Effect of TDX105 on the incidence and duration of HFSR of all grades

**FIGURE 2:**
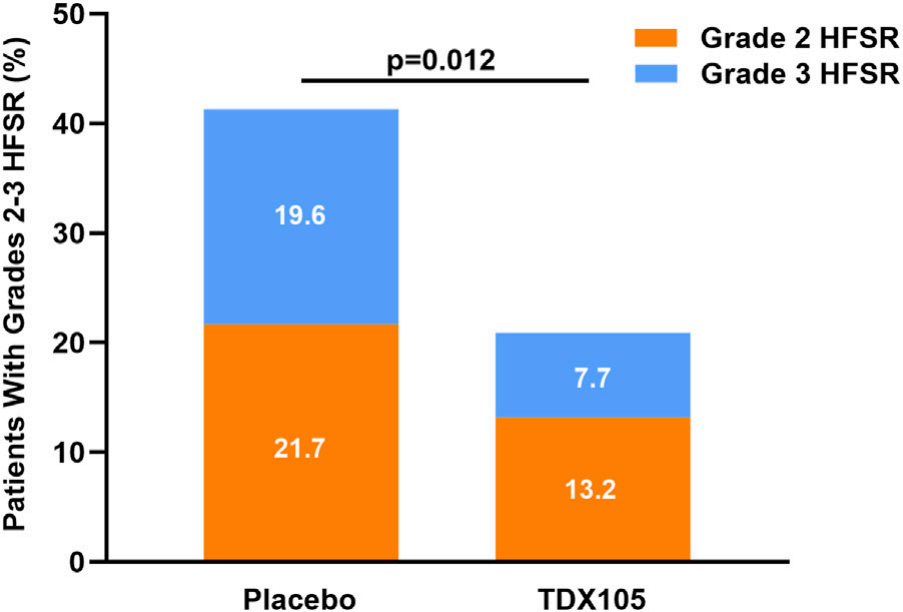
Occurrence of grade 2 or 3 HFSR in the experimental (TDX105) group and control (placebo) group within the 8-weeks treatment period.

After the conclusion of treatment, 84 patients had developed HFSR. The incidence of HFSR was 53.8% (n = 49) in the experimental group and 76.1% (n = 35) in the control group, with an ARD of 22.2% (*p* = 0.01, 95% CI: 0.1–0.4). The incidence of grade 1 HFSR specifically was 33.0% in the experimental group and 34.8% in the control group (*p* = 0.83, ARD: 1.82%, 95% CI: −0.1–0.2). For grade 2 HFSR, these rates were 13.2% and 21.7%, respectively (*p* = 0.19, ARD: 8.55%, 95% CI: −0.1–0.2), while for grade 3 HFSR, these rates were 7.7% and 19.6%, respectively (*p* = 0.04; ARD: 11.87%, 95% CI: −0.01–0.25). Together these results indicate that TDX105 significantly reduced the incidence and severity of Regorafenib-induced HFSR (Table 3), particularly grade 3 HFSR (Figure 2).

**TABLE 3:** Distribution of grades of HFSR.

HFSR cases, n (%)	TDX105 group (n = 91)	Placebo group (n = 46)	*p*-value
Grade 0	42 (46.2)	11 (23.9)	0.01
Grade 1	30 (33.0)	16 (34.8)	0.83
Grade 2	12 (13.2)	10 (21.7)	0.19
Grade 3	7 (7.7)	9 (19.6)	0.04

Abbreviation: HFSR, hand-foot skin reaction.

**FIGURE 3:**
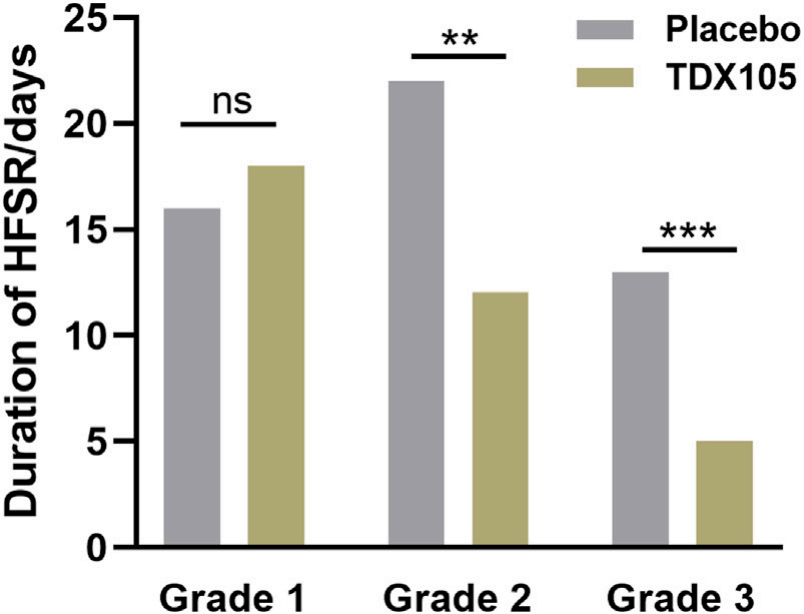
Duration of grades 1–3 HFSR in the experimental (TDX105) group and control (placebo) group.

Additionally, preventive intervention with TDX105 significantly delayed the onset of HFSR: the median time lag before HFSR onset was 25 days in the experimental group compared with 11 days in the control group (*p* < 0.001). Furthermore, the duration of HFSR in the experimental group was significantly shorter than that in the control group, especially for grade 2 HFSR (12 vs. 22 days, respectively, *p* = 0.0014) and grade 3 HFSR (5 vs. 13 days, respectively, *p* < 0.001; Table 4; Figure 3).

**TABLE 4:** Onset time and duration of HFSR.

HFSR	TDX105 group (n = 91)	Placebo group (n = 46)	*p*-value
Time of onset, days, median (IQR)	25 (18–35.75)	11 (8–15.5)	3.37E-10
Grade 1 duration	18 (14–23)	16 (11–22)	0.14
Grade 2 duration	12 (9.5–15)	22 (15–25.5)	1.39E-03
Grade 3 duration	5 (5–6.5)	13 (10–18)	8.78E-05

Abbreviation: HFSR, hand-foot skin reaction.

### Effect of TDX105 on the dose reduction and interruption of regorafenib

The final results revealed that the preventive intervention with TDX105 significantly reduced the rates of dose reduction and treatment interruption caused by Regorafenib-induced HFSR. Initially, the dose reduction rates for Regorafenib in the experimental and control groups were 32.26% and 39.13%, respectively (ARD: 6.87%, 95% CI: −0.1–0.2), and the interruption rates were 16.48% and 21.74%, respectively. The overall differences were not significant, and the rates of dose reductions and interruptions due to hypertension, fatigue, diarrhea, and other causes were not statistically significant. However, the rates of Regorafenib dose reduction and interruption due to HFSR did differ significantly between the two groups, with only 1 patient (1.01%) with dose reduction due to HFSR in the experimental group compared with 9 patients (19.57%) in the control group (ARD: 18.47%; 95% CI: 0.1–0.3, *p* < 0.05). Interruption of Regorafenib treatment due to HFSR occurred in no patients (0%) in the experimental group compared with 10.87% of patients in the control group (ARD: 10.87%; 95% CI: 0.02–0.2, *p* < 0.05; Tables 5, 6). These results indicate that the TCM TXD105 significantly improved patients’ compliance with Regorafenib treatment, thereby supporting optimal dosing in their oncology treatment plans.

**TABLE 5:** Causes of regorafenib dose reduction.

Cause of dose reduction, n (%)	TDX105 group (n = 91)	Placebo group (n = 46)
No dose reduction	58 (63.7)	28 (60.9)
Hand-foot skin reaction	1 (1.1)	9 (19.6)
Hypertension	10 (11.0)	5 (10.9)
Fatigue	8 (8.8)	4 (8.7)
Diarrhea	10 (11.0)	3 (6.5)

**TABLE 6:** Reasons for interruption of regorafenib.

Cause of regorafenib interruption, n (%)	TDX105 group (n = 91)	Placebo group (n = 46)
No dose interruption	76 (83.5)	36 (78.3)
Hand-foot skin reaction	0 (0.0)	5 (10.9)
Hypertension	2 (2.0)	0 (0.0)
Fatigue	6 (6.6)	2 (4.4)
Diarrhea	4 (4.4)	1 (2.2)

### Effect of TDX105 on PFS

Because this clinical trial primarily aimed to evaluate the mitigating effect of TDX105 on HFSR related to the targeted drug Regorafenib, there was no significant difference in treatment efficacy or the objective tumor response rate (ORR) between the two groups. No patients experienced complete remission of tumors. Partial response (PR) was observed in 3 cases in the experimental group and 2 cases in the control group. The disease control rates (DCRs) in the experimental and control groups were 58.24% and 50%, respectively (ARD: 8.24%, 95% CI: −0.1–0.3). Finally, the PFS was extended significantly in the experimental group to 3.2 months compared with the PFS of 2.5 months in the control group (*p* = 0.048). This difference may be due to the significant reduction in HFSR side effects caused by TDX105, which improved compliance with the Regorafenib treatment plan, ensuring normal implementation of the tumor treatment plan and contributing to the control of tumor progression.

### Exploratory analysis

**FIGURE 4:**
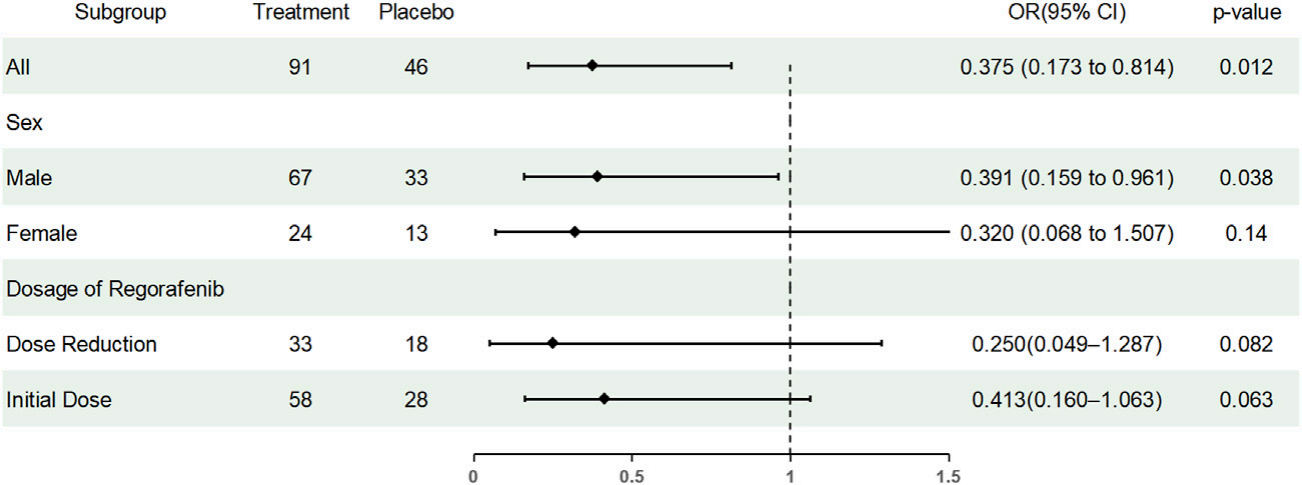
Forest plot showing the results of subgroup analyses. OR, odds ratio.

Subgroup analyses were conducted to assess whether the preventive effect of TDX105 on HFSR differed by sex or Regorafenib dose modification (Table 7). Among male patients, the incidence of HFSR was 52.2% in the experimental group versus 72.7% in the control group (OR = 0.41, 95% CI: 0.17–1.01, *p* = 0.05). Among female patients, the incidence was 58.3% versus 84.6% (OR = 0.26, 95% CI: 0.05–1.41). In patients without dose reduction, the incidence was 46.6% versus 67.9% (OR = 0.41, 95% CI: 0.16–1.06), and in those with dose reduction, it was 66.7% versus 88.9% (OR = 0.25, 95% CI: 0.05–1.29). The overall effect across the full cohort remained significant (OR = 0.37, 95% CI: 0.17–0.81). Logistic regression including interaction terms showed no significant modification of the preventive effect of TDX105 by sex or Regorafenib dose reduction status (Figure 4).

**TABLE 7:** Subgroup analysis by sex and dosage of regorafenib.

Subgroup	Absolute risk difference in incidence of HFSR, %	95% CI
All	−22.3	−38.1 to −6.5
Sex		
Male (n = 100)	−20.5	−39.8 to −1.2
Female (n = 37)	−26.3	−54.1 to +1.5
Dosage of regorafenib		
Dose reduction (n = 51)	−22.2	−43.9 to −0.6
Initial dose (n = 86)	−21.3	−42.8 to +0.2

Abbreviation: HFSR, hand-foot skin reaction.

### Effect of TDX105 on adverse events during regorafenib treatment

In this study, Regorafenib dose reduction was classified as an adverse event. During the study period, a total of 51 patients (37.23%) experienced adverse events, including 33 (36.26%) in the experimental group and 18 (39.13%) in the control group. The most common adverse events in the experimental group were hypertension and diarrhea (10.99% each), and that in the control group was grade 3 HFSR (19.57%). Throughout the study period, no cardiovascular event or death occurred in either group (Table 8).

**TABLE 8:** Adverse events in the TDX105 and placebo groups.

Adverse event, n (%)	TDX105 group (n = 91)	Placebo group (n = 46)
Any event	33 (36.3)	18 (39.1)
Hand-foot skin reaction	1 (1.1)	9 (19.6)
Hypertension	10 (11.0)	5 (10.9)
Fatigue	8 (8.8)	4 (8.7)
Diarrhea	10 (11.0)	3 (6.5)
Hepatic dysfunction	1 (1.1)	1 (2.2)
Proteinuria	2 (2.2)	1 (2.2)
Generalized rash	1 (1.1)	0 (0.0)
Perianal dermatitis	1 (1.1)	0 (0.0)
Mucositis	1 (1.1)	1 (2.2)

## Discussion

This study demonstrated significant efficacy of the TCM TDX105 in preventing HFSR associated with Regorafenib treatment. Treatment with TDX105 significantly reduced the overall incidence of HFSR, especially grade 3 HFSR, with consistent protective effects across subgroups, and also improved compliance with oncological treatment as well as PFS among patients with colorectal cancer receiving Regorafenib.

Despite the long-standing clinical issues related to HFSR and the availability of various treatment measures, current treatments for Regorafenib-associated HFSR typically involve symptomatic drugs such as moisturizing creams, urea or salicylic acid ointments, clobetasol propionate, corticosteroids, and topical antibiotics for anti-inflammatory and analgesic effects (McLellan et al., 2015). However, the existing treatment plans only alleviate symptoms and do not completely eliminate HFSR. Moreover, no unified treatment guideline has been generated to date. A randomized clinical trial in 2015 that evaluated the preventive effect of urea-based cream on sorafenib-induced HFSR found that patients treated with 10% urea-based cream plus best supportive care had a significantly lower total HFSR incidence within 12 weeks than the control group treated with only best supportive care (56.0% vs. 73.6%) (Ren et al., 2015). Moreover, the treatment significantly prolonged the time lag before the occurrence of the first HFSR. In addition, it may improve patients’ quality of life without compromising the efficacy of sorafenib treatment. However, the study did not incorporate a placebo control, and it had a non-blinded design. Thus, the study might have subjective bias, and the impact of urea cream on dose adjustment and interruption of sorafenib was not significant (Negri and Porta, 2015).

Subsequently, a multicenter, randomized, double-blind controlled trial also evaluated the preventive and enhancing effects of urea cream on HFSR in hepatocellular carcinoma patients treated with Sorafenib (Lee et al., 2020), in order to overcome the limitations of the aforementioned clinical studies, and its findings provided more robust evidence for clinical practice. The results of that study showed that the urea cream only significantly reduced the incidence of grade 2 or higher HFSR in the second week of treatment (from 23.9% in the placebo group to 13.8% in the urea cream group), but the difference in the overall incidence of HFSR was not statistically significant. Moreover, only 73 patients (25.32%) completed the 12-week follow-up in that study, precluding the collection of data on the long-term therapeutic effects. Nevertheless, the study also confirmed the potential applicability and favorable safety profile of urea cream for high-risk patients, suggesting that further larger-scale studies are warranted to fully evaluate its clinical efficacy. Moreover, another study (Naeem et al., 2024) found that topical urea did not significantly out-perform placebo in preventing HFSR in renal cell carcinoma patients treated with Sunitinib, and it had no significant impact on patients’ quality of life. This indicates that the reproducibility and stability of the preventive effect of urea cream may be poor.

In addition, the ReDOS trial (Jatoi et al., 2021) comparing the effects of preemptive and reactive topical clobetasol propionate on HFSR in patients treated with Regorafenib found that in two cycles, only 30% of patients in the clobetasol propionate prevention group did not experience HFSR. However, that study also did not incorporate a placebo control, and a high withdrawal rate and data loss in the second cycle may have affected the assessment of its long-term effects. In another study (Luo et al., 2020) involving *in vitro* and *in vivo* experiments, the SIRT1 inhibitor nicotinamide was found to alleviate sorafenib-induced HFSR. While that preliminary clinical study found that treatment with nicotinic acid (the pro-drug form of nicotinamide) reduced HFSR symptoms in patients with severe HFSR due to Sorafenib, the number of included patients was only 10. Therefore, further large-scale clinical trials are needed to verify these preliminary results.

The TCM TDX105 is an empirical prescription based on the principles of *clearing heat, drying dampness, cooling blood*, and *promoting blood circulation*. Clinically, it has been used for more than a decade for the topical treatment of skin injuries related to cancer therapy and has demonstrated significant clinical efficacy. Based on its clinical application, it rapidly alleviates inflammatory skin lesions characterized by redness, swelling, heat, pain, ulcers, and erosion. Our team, through preliminary research in network pharmacology, previously found that TDX105 may primarily exert its therapeutic effects through inflammation-related pathways such as PI3K-AKT and MAPK signaling (Li et al., 2022).

Previous research has shown that TDX105 demonstrates a significant advantage in efficacy over current conventional measures for treating HFSR (95.45% vs. 27.27%) (Tian et al., 2017). It is also convenient, safe, and effective for clinical use. The present study further explored the prophylactic effect of TDX105 on HFSR associated with Regorafenib treatment and found that the incidence of HFSR was reduced from 76.1% in the placebo control group to 53.8% in the TDX105 group. These results provide evidence that TDX105 significantly reduced the overall incidence of HFSR, especially grade 3 HFSR (7.7% vs. 19.6%) in the study patients while also delaying the onset time and reducing the duration of HFSR. The rates of dose reduction and treatment interruption due to Regorafenib-related HFSR also were significantly decreased with the use of TDX105, which may improve patients’ compliance with cancer treatment regimens and extend their PFS. The present study does have some limitations, such as a relatively small sample size, although it met the minimum required sample size calculated, thus giving the results statistical significance and reference value. In addition, this study included only Chinese patients, and therefore, the ethnic and geographic generalizability of the results requires further validation. In our future studies, we will incorporate quality-of-life assessments, which can provide valuable functional data and further enhance the clinical relevance of the findings. Furthermore, we will consider including a reference treatment group in subsequent trials to strengthen the robustness of comparative analyses and to support more definitive conclusions. To make the study results more comprehensive and authoritative, our team plans to conduct larger-scale clinical trials in the future and to design *in vitro* and *in vivo* experiments to explore the pharmacological mechanisms responsible for the effectiveness of this TCM.

## Conclusion

The results of this study showed that the TCM TDX105, when used topically as a preventive intervention, reduced the incidence and severity of HFSR, with rates of 53.8% (n = 49) in the experimental group versus 76.1% (n = 35) in the control group (ARD = 22.2%; *p* = 0.01). In particular, the incidence of grade 3 HFSR was lower in the TDX105 group (7.7% vs. 19.6%, *p* = 0.04). Additionally, preventive intervention with TDX105 significantly delayed the onset of HFSR (25 days vs. 11 days, *p* < 0.001) and shortened its duration, especially for grade 2 HFSR (12 vs. 22 days, *p* = 0.0014) and grade 3 HFSR (5 vs. 13 days, *p* < 0.001). Furthermore, TDX105 improved compliance with Regorafenib treatment among colorectal cancer patients, as reflected by prolonged PFS (95 days vs. 74.5 days, *p* = 0.048), with consistent protective effects across subgroups. Therefore, TDX105 may be a suitable preventive intervention for skin lesions associated with Regorafenib treatment-related HFSR, which will benefit the implementation of optimal dosing in cancer treatment regimens.

## Data Availability

The raw data supporting the conclusions of this article will be made available by the authors, without undue reservation.

## Abbreviations

95% CI, 95% confidence interval; CTCAE, Common Terminology Criteria for Adverse Events; DCR, disease control rate; HFSR, hand–foot skin reaction; MAPK, mitogen-activated protein kinase; OR, odds ratio; ORR, objective tumor response rate; PR, partial response; PI3K-AKT, phosphatidylinositol-3-kinase protein kinase B; RECIST, Response Evaluation Criteria in Solid Tumors; TCM, traditional Chinese medicine.
